# Knockout of a papain-like cysteine protease gene *OCP* enhances blast resistance in rice

**DOI:** 10.3389/fpls.2022.1065253

**Published:** 2022-11-30

**Authors:** Yuying Li, Pengcheng Liu, Le Mei, Guanghuai Jiang, Qianwen Lv, Wenxue Zhai, Chunrong Li

**Affiliations:** ^1^ Institute of Genetics and Developmental Biology, Chinese Academy of Sciences, Beijing, China; ^2^ University of Chinese Academy of Sciences, Beijing, China; ^3^ College of Chemistry and Life Sciences, Zhejiang Normal University, Jinhua, China

**Keywords:** blast resistance, oryzain alpha chain precursor (OCP), OsRACK1A, OsSNAP32, papain-like cysteine proteases (PLCPs), rice (*Oryza sativa*)

## Abstract

Papain-like cysteine proteases (PLCPs) play an important role in the immune response of plants. In Arabidopsis, several homologous genes are known to be involved in defending against pathogens. However, the effects of PLCPs on diseases that afflict rice are largely unknown. In this study, we show that a PLCP, an oryzain alpha chain precursor (OCP), the ortholog of the Arabidopsis protease RD21 (responsive to dehydration 21), participates in regulating resistance to blast disease with a shorter lesion length characterizing the knockout lines (*ocp-ko*), generated *via* CRISPR/Cas9 technology. OCP was expressed in all rice tissues and mainly located in the cytoplasm. We prove that OCP, featuring cysteine protease activity, interacts with OsRACK1A (receptor for activated C kinase 1) and OsSNAP32 (synaptosome-associated protein of 32 kD) physically *in vitro* and *in vivo*, and they co-locate in the rice cytoplasm but cannot form a ternary complex. Many genes related to plant immunity were enriched in the *ocp-ko1* line whose expression levels changed significantly. The expression of jasmonic acid (JA) and ethylene (ET) biosynthesis and regulatory genes were up-regulated, while that of auxin efflux transporters was down-regulated in *ocp-ko1*. Therefore, OCP negatively regulates blast resistance in rice by interacting with OsRACK1A or OsSNAP32 and influencing the expression profiles of many resistance-related genes. Moreover, OCP might be the cornerstone of blast resistance by suppressing the activation of JA and ET signaling pathways as well as promoting auxin signaling pathways. Our research provides a comprehensive resource of PLCPs for rice plants in defense against pathogens that is also of potential breeding value.

## Introduction

The most destructive disease of cultivated rice is blast, caused by the fungus *Magnaporthe oryzae* (Zhai et al., 2022), which results in severe yield losses of about 30% (Yin et al., 2021). To defend against pathogens, plants have evolved complex immune systems, one is the basal defense defined by pattern recognition receptors, and the other is the immune response mediated by *Resistance* (*R*) genes (Yu et al., 2021). It is widely accepted that generating rice germplasm resources with *R* genes is the most economical and eco-friendly strategy to defend crops against blast (Xiao et al., 2020). Therefore, it is imperative we discover new *R* genes for controlling blast disease. Some blast-resistance genes have been cloned already and applied in crop breeding for disease resistance (Li et al., 2020). For example, *Pita* (*pyricularia oryzae resistance-ta*), encoding a major *R*-gene type, confers resistance to *M. oryzae* races containing the corresponding avirulence gene *AVR-Pita* (Bryan et al., 2000). The *pi21* (*pyricularia oryzae resistance 21*), encoding a proline-rich protein, is a non-race specific recessive gene that maintains resistance permanently, although this trait is incomplete in comparison with that triggered by *R* genes (Fukuoka et al., 2009; Nawaz et al., 2020; Tao et al., 2021). Moreover, there are some non-*R* genes in rice that can participate in blast resistance. Transgenic lines featured increased blast resistance when the SNAP25-type gene *OsSNAP32* was overexpressed, which encodes a soluble N-ethylmaleimide-sensitive-factor attachment protein receptor, whose expression is induced by blast fungus inoculation in rice seedlings (Bao et al., 2008; Luo et al., 2016). OsSYP121 (i.e., syntaxin of plants 121) can interact with OsSNAP32 and VAMP714/724 (vesicle-associated membrane protein 714/724) to form a complex that mediated host resistance to rice blast (Cao et al., 2019). Besides, many transcription factors and enzymes also are involved in blast resistance, such as *OsWRKY45* (*WRKY gene 45*) (Shimono et al., 2007) and *OsPAL1* (*phenylalanine ammonia-lyase 1*) (Zhou et al., 2018), to name two. Nevertheless, due to the diversity and complexity of pathogenic populations, it is difficult for rice blast resistance-related genes to effectively maintain their resistance or, if they do, it only applies to limited regions (Tao et al., 2021). Accordingly, it is necessary to continuously uncover more *R* genes to blast for rice crop improvement.

Cysteine proteases function critically in plant growth and development, of which the PLCPs are notable for being involved in protein maturation and degradation, plant senescence, seed germination, and programmed cell death (PCD) (Grudkowska and Zagdanska, 2004; Liu et al., 2018). Moreover, PLCPs play key roles in plant immune systems by inducing systemic immunity and degrading the pathogen-effector protein (Perez-Lopez et al., 2021). For example, in Arabidopsis, the *rd21a* mutants were more susceptible to the fungal pathogen *Botrytis cinerea* (Shindo et al., 2012), and knocking out *rd21a* inhibited flg22-triggered stomatal closure, which led to lowered resistance to *Pseudomonas syringae* (Liu et al., 2020). Null *XCP1* (*xylem cysteine peptidase 1*) or *XCP2* (*xylem cysteine peptidase 2*) mutants display increased resistance to pathogens (Zhang et al., 2014; Perez-Lopez et al., 2021). In tomato, C14/CYP1, targeted by the *Phytophthora infestans* effector AvrBlb2, plays a role in pathogen defense in that silencing *C14* increased susceptibility to *P. infestans* (Kaschani et al., 2010; Misas-Villamil et al., 2016). For rice, there are few reports about how PLCPs affect its growth and development. *OsCP1* (*cysteine protease 1*), a cysteine protease gene, was shown to influence pollen development and regulate PCD (Lee et al., 2004; Li et al., 2011). Yet whether and how the PLCPs function in rice immunity remains unclear.

Here we identified a PLCP, an oryzain alpha chain precursor (OCP), which is capable of negatively regulating rice blast resistance. Knocking out *OCP* resulted in the accumulation of mRNA for defense-related genes and shortened the lesion length of transgenic plants (*ocp-ko*) inoculated with blast isolates when compared with TP309. We find that *OCP* is highly conserved in plants and possesses the cysteine protease activity. By screening a yeast library, two rice proteins related to blast resistance were obtained, namely OsRACK1A and OsSNAP32. Many blast resistance genes are up-regulated in *ocp-ko* plants, which meant that OCP probably negatively regulates blast resistance by repressing the related gene expression. Therefore, this study not only fills the knowledge gap of PLCPs in disease resistance of rice but also provides effective and promising gene resources for use in future rice breeding.

## Materials and methods

### Plant materials and growth conditions

The *Japonica* rice cultivar TP309 was used for the transgenic experiments. All plants were cultivated in the experimental field of the Institute of Genetics and Developmental Biology, Chinese Academy of Sciences. To generate gene overexpression, the coding sequence (CDS) of *OCP* was amplified from the cDNA of TP309 and cloned into the vector UBI-pCAMBIA1300; the knockout mutants were created using the CRISPR/Cas9 system. All the constructs were transformed into rice calli *via Agrobacterium*-mediated transformation.

### Structural analysis and construction of the phylogenetic tree of orthologous proteins

Amino acid sequences of the plant species and corresponding accession numbers were retrieved from the NCBI (https://www.ncbi.nlm.nih.gov/), RGAP (http://rice.uga.edu/index.shtml), and WheatOmics 1.0 (http://wheatomics.sdau.edu.cn/). The structures of orthologous proteins were drawn with SMART (http://smart.embl-heidelberg.de/). Based on the alignment of the amino acid sequences with the Muscle program, and using 1000 bootstrap replicates, a neighbor-joining tree was constructed in MEGA7 software. Multiple sequence alignment of the proteins was carried out by MEGA7 and the results were edited by GeneDoc software.

### Blast inoculation assays

For the blast fungal spray inoculation assays, the plants were grown in a greenhouse at 28°C under a 12-h light/12-h dark photoperiod for 14 days. The assay was performed as described by Chen et al. (2018). The spore concentration was adjusted to 1 × 10^5^ cfu/mL with 0.2% Tween-20, and the inoculated rice seedlings were kept in a dark chamber at 28°C for 24 h, and then moved into the greenhouse. The injection method for testing blast resistance in the field was followed (Lv et al., 2013). The seedlings at the tillering stage were injected with spore suspension, and the leaf status was observed 7 days after inoculation.

For the punch inoculation assays, 40-day-old rice plants were inoculated by following a previously described methodology (Park et al., 2012), albeit with slight modifications. The rice leaves were lightly wounded with 10-μL pipette tips on a 1.5-cm scale and put on the surface of 6-Benzylaminopurine, and the spore suspension was added onto the wound site. The ensuing lesion length was measured at 8 days post-inoculation in the greenhouse. The *M. oryzae* isolates 97-27-2, JL021605, and ZB13 were used in this study.

### GUS staining

GUS activity was analyzed in transgenic plants ***via*** histochemical staining with 5-bromo-4-chloro-3-indolyl-b-Dglucuronicacid (X-Gluc), as described previously (Dong et al., 2021). The rice tissues were incubated for 16 h at 37°C in a staining buffer (100 mM sodium phosphate [pH 7.0], 10 mM EDTA, 0.5 mM K_4_Fe(CN)_6_, 0.5 mM K_3_Fe(CN)_6_, 0.1% [v/v] Triton X-100, and 1 mM X-Gluc), and then decolorized in 100% ethanol before photographing them.

### Rice protoplast preparation and transformation

Rice protoplasts were prepared from 2-week-old seedlings of TP309 that had been grown in darkness. Protoplasts were transformed as described previously (Bart et al., 2006). Plasmid constructs were transformed into the rice protoplasts, which were then kept at 28°C for 16 or 18 h. After that, we detected the fluorescence or extracted proteins.

### Subcellular localization

The coding region of *OCP* was fused with the green fluorescent protein (35S::OCP-GFP) and enhanced yellow fluorescent protein (35S::OCP-eYFP), and then transformed into TP309 calli and rice protoplasts to express the fusion proteins, respectively. For subcellular co-localization, we fused the coding region of OsRACK1A or OsSNAP32 with the mCherry tag and then transformed them into rice protoplasts alone or with 35S::OCP-eYFP. The fluorescent signal was visualized using the Zeiss LSM 710 NLO microscope (Carl Zeiss, Oberkochen, Germany) after incubation at 28°C for 16 h.

### Yeast hybrid assays

The coding region of *OCP* was introduced into the pGBKT7 vector (BD-OCP) as bait and co-transformed with the rice cDNA library for the screening of interacting proteins on SD/-Leu-Trp-His-Ade selected plates. For specific interactions, the truncations of *OCP* were cloned into the pGBKT7 vector and the full-length coding region of *OsRACK1A* and *OsSNAP32* were cloned into the pGADT7 vector (respectively yielding AD-OsRACK1A and AD-OsSNAP32), and then co-transformed into the yeast strain Gold Y2H. The transformants were grown on SD/-Trp-Leu medium at 30°C for 3 to 5 days and the interaction was confirmed by colony growth on SD/-Ade-His-Leu-Trp (AD/-A-H-L-T) medium with X-α-gal.

For the yeast three-hybrid (Y3H) assays, the coding region of *OCP* was cloned into the multiple cloning site (MCS) 2 of pBridge (pBridge-OCP), and the coding region of *OsSNAP32* was introduced into MCS1 of pBridge (pBridge-OsSNAP32 and pBridge-OsSNAP32-OCP). Each of these vectors was then co-transformed into Gold Y2H with AD-OsRACK1A. The transformants were grown on (SD)/-Met-Trp-Leu medium at 30°C for about 5 days, and the interaction was confirmed by colony growth on SD/-Ade-His-Leu-Met-Trp (SD/A-H-L-M-T) medium with X-α-gal for about 5 days. Specific primers used are listed in **Supplementary Table 1**.

### Bimolecular fluorescence complementation assays

The full-length coding region of *OCP* was cloned into the pVYCE vector (cYFP-OCP), and OsRACK1A and OsSNAP32 were introduced into the pVYNE vector (nYFP-OsRACK1A and nYFP-OsSNAP32). The ensuing constructs were transformed into the *Agrobacterium tumefaciens* strain EHA105, and then allowed to infect 5-week-old *Nicotiana benthamiana* leaves. Fluorescent signals were detected and photographed using Zeiss LSM 710 NLO microscope (Carl Zeiss, Oberkochen, Germany) after infiltration for 3 or 4 days (Cao et al., 2019).

### Co-immunoprecipitation assays

To verify the interaction of OCP with OsRACK1A and OsSNAP32 *in vivo*, the recombinant vectors OCP-Myc, OsRACK1A-mCherry, and OsSNAP32-mCherry were generated. These construct pairs were transiently co-expressed in rice protoplasts. After 18 h, the protoplasts were collected by centrifugation at 150 × *g* and rinsed three times with a wash buffer (50 mM Tris-HCl [pH 7.5], 150 mM NaCl). The total proteins were extracted using a lysis buffer (50 mM Tris-HCl [pH 7.5], 150 mM NaCl, 0.5% Triton X-100, 0.5% Nonidet P-40 [NP-40], 1 μM MG132, and 1 × Protein Inhibitor Cocktail). After its 1-h incubation on ice, the lysate was centrifuged at 4°C with 15 000 × *g*. Lysates containing the target proteins were incubated with 20 μL of Myc tag-Nanoab-Agarose Beads by tumbling them for 2 h at 4°C. Next, the beads were rinsed thrice with the wash buffer and boiled for 5 min with an SDS loading buffer. The proteins were analyzed by running a Western blot assay using anti-Myc and anti-mCherry.

### Cysteine protease activity profiling

Procedures for cysteine protease activity profiling were largely followed as described previously (van der Hoorn et al., 2004). L-cysteine, E-64, and DCG-04 were purchased from Lablead (Beijing, China), Bioss (Beijing, China), and MedKoo (Morrisville, USA), respectively. Proteins were extracted from *Escherichia coli* and purified. About 30 μg of protein was mixed with 50 mM sodium acetate buffer (pH 6), to which was added 10 mM L-cysteine and 2 μM DCG-04; 0.4 mM E-64 was held in another tube as the control. The samples were incubated at room temperature for 5 h. Then proteins were precipitated by adding 1 mL of ice-cold acetone and collected by centrifugation (for 1 min, at 10 000 × *g*). Proteins were washed twice with 70% acetone and dissolved in 30 μL of TBS, boiled in 30 μL of SDS sample buffer, and finally separated on 10% SDS gels.

### Protein stability tests

EGFP-His, GST-OsRACK1A, and GST-OsSNAP32 were extracted from *E. coli*. The proteins of the same quality were mixed and treated at room temperature for 30 min and 60 min. The fusion protein was detected by the corresponding antibody.

### Transcriptome analysis

Total RNA was extracted from *ocp-ko1* and TP309 for transcriptome sequencing. The Volcano plot and KEGG enrichment analyses were completed using Majorbio (https://cloud.majorbio.com/). Heatmaps were generated using TBtools.

### Total RNA isolation and qRT-PCR analysis

Total RNA was extracted using the TRIzol reagent (Invitrogen, Waltham, MA, USA) by following the manufacturer’s protocol, after which cDNA was synthesized using the ReverTra Ace 1 qPCR RT Master Mix with gDNA Remover (TOYOBO, Osaka, Japan). The qRT-PCR was performed using 2 × T5 Fast qPCR Mix (SYBR Green I, Tsingke, Beijing, China) according to the manufacturer’s instructions. The rice *OsActin* gene served as an internal control for the data normalization in the formal analysis. The results are presented as the mean ± SD in triplicate. Bar graphs were generated using GraphPad Prism 9. The specific primers used are listed in **Supplementary Table 2**.

## Results

### Structure and evolution analysis of OCP and its orthologous proteins in various plant species

**Figure 1:**
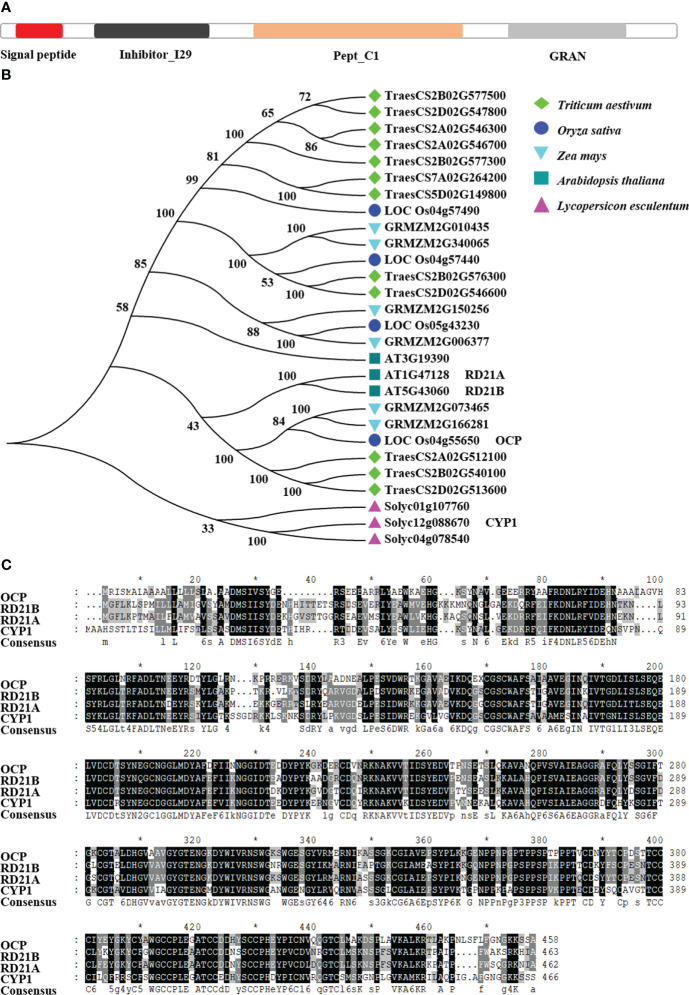
Evolutionary analysis of OCP and its orthologous proteins. **(A)** Structures of OCP. **(B)** Phylogenetic tree of OCP and its orthologous proteins in five plant species. Based on a shared amino acid sequence identity greater than 50% with OCP, 27 amino acid sequences were collected from NCBI. The neighbor-joining tree was built using the Muscle program, with 1000 bootstrap replicates, in MEGA7. Protein structure was analyzed by SMART. All the proteins contained the same four domains, signal peptide, inhibitor I29, pept_C1, and GRAN. **(C)** Comparison of the amino acid sequences of OCP with RD21B, RD21A, and CYP1. The black coloring shows the same amino acids present in the four proteins. The * indicates the amino acid position number, 10, 30, 50......

The OCP contains four main domains, namely signal peptide, inhibitor I29, pept_C1, and GRAN (**Figure 1A**). The signal peptide started at position 1 aa (amino acid) and ended at position 21 aa. Inhibitor I29 was the cathepsin propeptide inhibitor domain (40–100 aa). Pept_C1 was the enzyme active domain, belonging to the papain family of cysteine proteases (129–344 aa). GRAN, granulin, is probably released by post-translational proteolytic processing carried out at 367 to 424 aa. Based on the consistency of amino acid sequence with OCP of at least 50% and all above four domains being present, we selected 28 amino acid sequences of five species to build a phylogenetic tree, as shown in **Figure 1B**. There were three orthologous proteins of OCP in rice, of which OsCP1 was reported to affect pollen development, seed germination, and plant height (Lee et al., 2004; Li et al., 2011). In *A. thaliana*, there were three proteins, including RD21B and RD21A, whose sequence alignment consistency with OCP was 68.13% and 67.59%, respectively (**Figure 1C**). Of three orthologous proteins in tomato, only CYP1 has been studied (Kaschani et al., 2010; Misas-Villamil et al., 2016), and its sequence alignment consistency with OCP was 62.5%. Six and 12 proteins were found in maize and wheat, respectively. Hence, we concluded OCP is highly conserved in plants.

### Knockout of *OCP* enhances rice blast resistance

**Figure 2:**
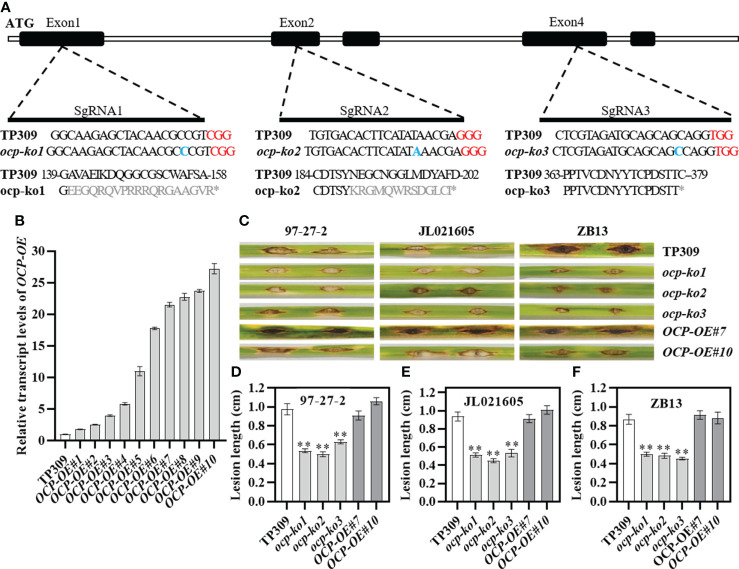
Mutant types and blast resistance identification of OCP. **(A)** Three kinds of allelic variations induced by CRISPR/Cas9 in different regions. CDS are shown in the black boxes. Untranslated regions (UTR) and introns correspond to the white sections. The sgRNA1, sgRNA2, and sgRNA3 were located in Exon 1, Exon 2, and Exon 4, respectively, corresponding to three domains of OCP, inhibitor I29, pept_C1, and GRAN. Red bases indicate the protospacer adjacent motif (PAM) recognition sites. Blue bases show the insert location. In gray are the altered amino acid residues due to mutation. The * indicates the terminate codon. **(B)** Relative transcript levels of 10 overexpression lines (*OCP-OE#1* to *OCP-OE#10*) in T1 of *OCP*. **(C)** Punch inoculation of wild-type rice TP309 and *OCP* mutant lines with the blast isolate 97-27-2, JL021605, and ZB13. This experiment was repeated twice. **(D–F)** Lesion lengths (mean ± SEM, n ≥ 8) of the tested lines according to the results in **(C)**. Asterisks indicate statistical significance compared with TP309 (** P ≤ 0.01, *t* test).

In order to explore the specific effects of *OCP* on rice growth and development, we constructed *OCP* knockout vectors with the sgRNA located at the first, second, and fourth exon, these distributed in the inhibitor I29, pept_C1, and GRAN domains, respectively, by using the CRISPR/Cas9 genome editing approach. The *OCP* overexpression vector was driven by the 35S promoter. These vectors were transformed into TP309 calli *via Agrobacterium*-mediated transformation. Through screening, 20, 11, and 5 edited plants of T0 progeny were obtained for three editing sites, respectively. Next, we found that the *ocp-ko* plants were mainly characterized by insertion or deletion of one or more bases. Most editing forms consisted of a single base insertion, and the insertion sites were unified. These mutant types led to an open-reading frame code shift for *OCP* and the premature termination of its translation (**Figure 2A**). To identify the overexpressing plants, we conducted quantitative real-time PCR (qRT-PCR) assays, by extracting total RNA from the T1 leaf. These results confirmed there were differences in expression levels among individual lines, with the highest level found for *OCP-OE#10* (~27-fold change) and the lowest level for *OCP-OE#1* (~1.8-fold change). Five independent transgenic lines had an expression level of *OCP* that was 15 times greater than that of wild-type plants (**Figure 2B**). Finally, *ocp-ko1*, *ocp-ko2*, *ocp-ko3*, *OCP-OE#7*, and *OCP-OE#10* lines were chosen for further study.

Cysteine proteases participate in plant immune responses (Shindo et al., 2012; Ormancey et al., 2019). To clarify the response of *OCP* to pathogens, we inoculated the wild-type TP309, *ocp-ko1*, *ocp-ko2*, *ocp-ko3*, and *OCP-OE#10* with six *Xanthomonas oryzae* pv*. oryzae* (*Xoo*) isolates. These results revealed no significant difference in disease among these tested plants (**Supplementary Figure 1**). Thus *OCP* was not involved in the resistance to bacterial blight. Then we inoculated these lines with *M. oryzae* isolates (97-27-2, JL021605, and ZB13) to assess their resistance to blast. Compared with TP309, the *ocp-ko* mutants exhibited a significantly shorter lesion length (**Figure 2C**). For all inoculated *M. oryzae* isolates, there was no significant size difference between the lesions of TP309 and *OCP-OE lines*, but those of *ocp-ko* lines were significantly reduced. For example, *ocp-ko1* plants had 0.54 ± 0.02 cm, 0.51 ± 0.02 cm, and 0.50 ± 0.02 cm lesions, corresponding to the three isolates, while TP309 had 0.98 ± 0.06 cm, 0.94 ± 0.05 cm, and 0.87 ± 0.05 cm, respectively (**Figures 2D–F**).

Then we validated the resistance to blast of the tested lines (TP309, *ocp-ko1*, and *OCP-OE#10*), by conducting spray inoculation assays. The lesion numbers of *ocp-ko1* were dramatically reduced with isolates 97-27-2, JL021605, and ZB13, whereas they were much more abundant for TP309 and *OCP-OE#10* (**Supplementary Figure 2**). In addition, we inoculated TP309, *ocp-ko1*, and *OCP-OE#10* with JL021605 in the field, finding that *ocp-ko1* plants featured a shorter lesion length when compared to TP309 (**Supplementary Figure 3**). Altogether, these results show that the *OCP* knockout enhances the resistance to blast but not bacterial blight in rice.

### Expression pattern and protease activity analysis of OCP

**Figure 3:**
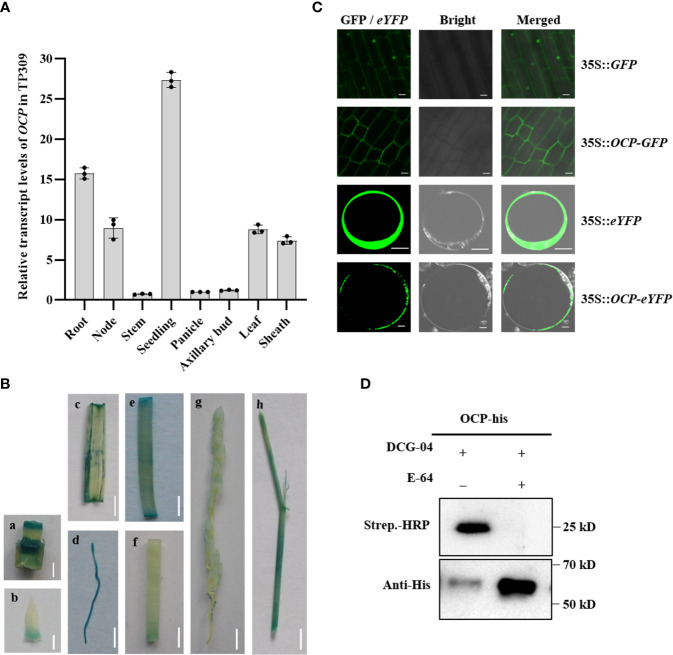
Expression pattern and cysteine protease activity analysis of OCP. **(A)** *OCP* RNA expression pattern obtained by qRT-PCR in rice TP309 (mean ± SEM, n = 3). **(B)** GUS activity of *OCP* in different rice tissues. a, node; b, axillary bud; c, leaf; d, root; e, sheath; f, stem; g, panicle; h, leaf and sheath of seedlings. Scale bars: a = 5 mm; b = 2 mm; c–h = 10 mm. **(C)** Subcellular localization of OCP in rice root tips and rice protoplasts. Scale bar = 10 μm. **(D)** Protease activity profiling of OCP *in vitro*. E-64 was an effective inhibitor of cysteine proteases, and DCG-04 was a biotinylated derivative of E-64. The biotinylated protease could be detected on a protein gel blot using a conjugate of streptavidin with HRP (strep.-HRP). OCP-His was detected with Anti-His.

The temporal and spatial expression pattern of the *OCP* gene was investigated in different tissues of TP309 by qRT-PCR. The gene was expressed in rice various tissues examined, albeit at a higher level in the seedling and at lower levels in the stem, panicle, and axillary bud, and at intermediate levels in the root, node, leaf, and sheath (**Figure 3A**). To further confirm the expression levels of *OCP*, transgenic plants were generated with the expression of a β-glucuronidase (GUS) driven by the promoter of *OCP* in TP309. Strong GUS activity was detected in the root, leaf, and sheath of seedling (**Figure 3B**), supporting well the qRT-PCR results.

To determine the subcellular localization of OCP, we fused the *OCP* coding region with the green fluorescent protein (GFP) and the enhanced yellow fluorescent protein (eYFP) driven by the cauliflower mosaic virus 35S promoter at the C terminus and expressed the fusion proteins in rice plants and protoplasts. Laser confocal microscopy revealed that the signal of the GFP-tagged OCP protein was excluded from the nucleus; meanwhile, the GFP signal alone was expressed in both the nucleus and cytoplasm in the roots of transgenic plants. In rice protoplasts, the green fluorescence emitted by the fusion protein was detected exclusively in the cytoplasm (**Figure 3C**). Therefore, OCP is located in the cytoplasm.

As the orthologous gene of *RD21*, *OCP* encodes a cysteine protease; hence, we further profiled protease activity by Western blotting *in vitro*. The method used followed one describe before (van der Hoorn et al., 2004). DCG-04 is a biotinylated derivative of the E-64 cysteine protease inhibitor. Active cysteine protease cleaves protein substrates through a covalent intermediate state, in that the biotinylation of active proteases by DCG-04 occurs because the cleavage mechanism is locked in a covalent intermediate state. Biotinylated proteases can be detected on SDS-PAGE gel using streptavidin conjugated to HRP. Firstly, the protein of OCP was expressed in *E. coli*, and we found that the protein mainly existed in the supernatant and only a small part occurred in the sediment (**Supplementary Figure 4**), which facilitated collection of the target protein. Then we treated the same content protein with DCG-04 and E-64 (as a control). These results showed that it was detected with streptavidin-HRP when OCP was treated with DCG-04 alone, and excess E-64 inhibited the binding of DCG-04 and OCP (**Figure 3D**). In addition, more protein remained in the sample with E-64 detected with anti-His. Taken together, our results demonstrated OCP has cysteine protease activity, and that E-64 can effectively delay the degradation of OCP.

### OCP physically interacts with OsRACK1A and OsSNAP32

**Figure 4:**
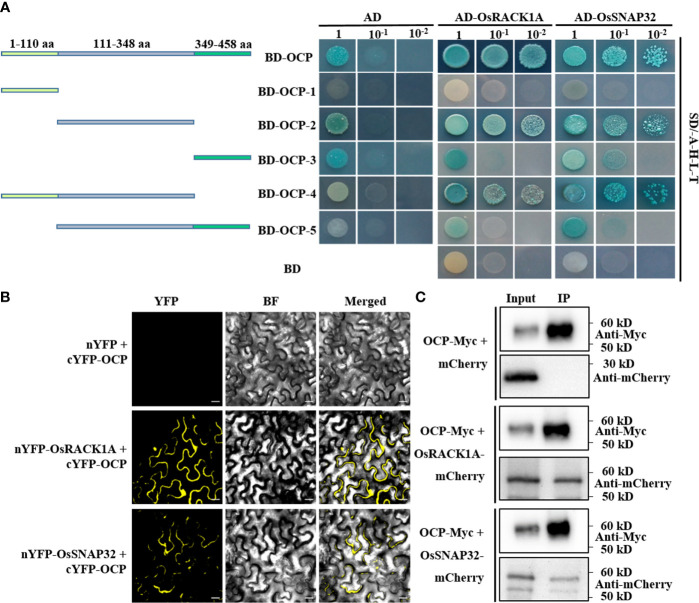
OCP physically interacts with OsRACK1A or OsSNAP32. **(A)** OCP and its truncations interact with OsRACK1A or OsSNAP32 in Y2H. AD, pGADT7; BD, pGBKT7; BD-OCP, full length of OCP; BD-OCP-1, 1–110 amino acid (aa) of OCP; BD-OCP-2, 111–348 aa; BD-OCP-3, 349–458 aa; BD-OCP-4, 1–348 aa; BD-OCP-5, 111–458 aa. 20 mM 3-amino-1,2,4-triazole (3-AT) was added to BD-OCP-3 and BD-OCP-5. **(B)** BiFC assay showing the interaction of OCP with OsRACK1A or OsSNAP32 in tobacco leaf epidermal cells. nYFP+cYFP-OCP was the negative control. Bar = 20 μm. **(C)** Co-immunoprecipitation assays to verify the interaction of OCP with OsRACK1A or OsSNAP32 in rice protoplasts. OCP-Myc+mCherry was the negative control. OsRACK1A-mCherry and OsSNAP32-mCherry were detected with Anti-mCherry.

To identify the interaction partners of OCP, we performed a yeast two-hybrid (Y2H) screen using a cDNA library of rice. The coding region of *OCP* was cloned into the bait vector (BD-OCP), and no autoactivation and toxicity of OCP were proven (**Supplementary Figure 5**). Then, BD-OCP was co-transformed with a cDNA library into yeast cells. Forty-four clones were isolated from the quadruple dropout media (-L-T-H-A) (**Supplementary Table 3**), and their sequences were analyzed *via* amplifying and sequencing. Two possible interacting proteins, OsRACK1A and OsSNAP32, participating in rice blast resistance (Nakashima et al., 2008; Luo et al., 2016), were thus obtained. Next, the CDS of *OsRACK1A* and *OsSNAP32* were fused to the GAL4 activation domain (AD-OsRACK1A and AD-OsSNAP32), and each co-transformed with BD-OCP into yeast cells. These cells grew well on the quadruple dropout media containing BD-OCP and AD-OsRACK1A or AD-OsSNAP32, while those transformed with the corresponding control did not (**Figure 4A**). To clarify the domains interacting with OsRACK1A or OsSNAP32, we divided the protein of OCP into five segments (**Figure 4A**). Interestingly, BD-OCP-3 and BD-OCP-5 interacted with the empty vector AD, and 20 mM 3-AT was then used to verify the actual interaction (**Supplementary Figure 6**). Lastly, we found that BD-OCP-2 and BD-OCP-4 grew well on the screening medium with AD-OsRACK1A or AD-OsSNAP32, which demonstrated that pept_C1 was effective for their interaction.

To further verify the interaction between OCP and OsRACK1A or OsSNAP32, we conducted a bimolecular fluorescence complementation (BiFC) assay to produce the fusion proteins cYFP-OCP and nYFP-OsRACK1A or nYFP-OsSNAP32 in *N. benthamiana* leaves *via Agrobacterium*-mediated transformation. These results showed that OCP interacted with OsRACK1A or OsSNAP32, with the associated fluorescence detected in the cytoplasm predominantly (**Figure 4B**). Meanwhile, co-immunoprecipitation (Co-IP) assays to test the interaction *in vivo* were carried out, co-expressing the fusion proteins OCP-Myc and OsRACK1A-mCherry, OsSNAP32-mCherry, or mCherry alone in the rice protoplasts. A band was detected for OCP-Myc and OsRACK1A-mCherry or OsSNAP32-mCherry in their IP sample, but no band was discernible in the OCP-Myc and mCherry IP samples when using an anti-mCherry antibody (**Figure 4C**). Together, these results suggested that OCP interacts with OsRACK1A or OsSNAP32 physically, *in vitro* and *in vivo*.

### OsRACK1A and OsSNAP32 cannot form a complex *via* OCP

**Figure 5:**
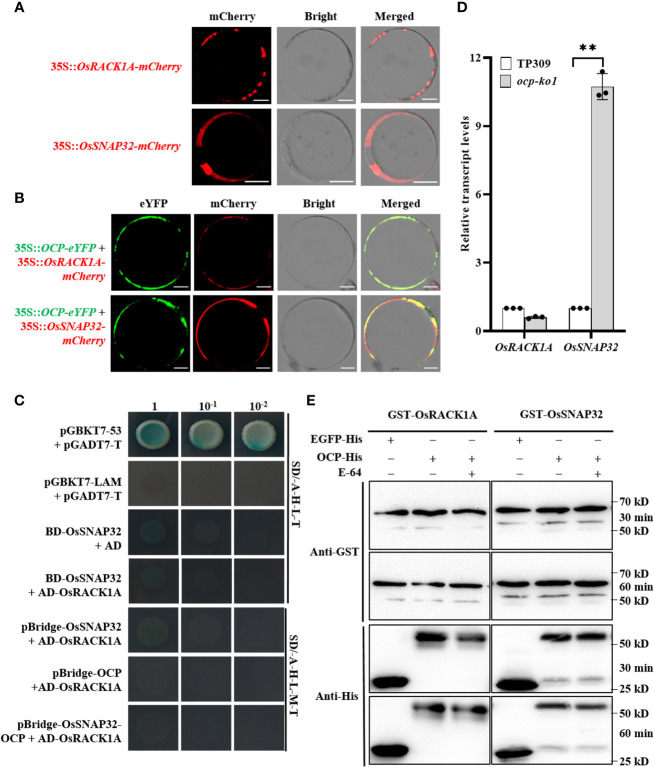
OCP is unable to form a complex with OsRACK1A or OsSNAP32, or affect the stability of either. **(A)** Subcellular localization of OsRACK1A and OsSNAP32 in rice protoplasts. Scale bar = 10 μm. **(B)** Subcellular co-localization of OCP and OsRACK1A or OsSNAP32 in rice protoplasts. Scale bar = 10 μm. **(C)** Yeast two-hybrid assays showing the non-interaction of OsRACK1A and OsSNAP32; pGBKT7-53 and pGADT7-T were used as positive controls, while pGBKT7-Lam and pGADT7-T were used as negative controls. Yeast three-hybrid assays showed that OCP, OsRACK1A, and OsSNAP32 could not form a complex. **(D)** Relative transcript levels of OsRACK1A and OsSNAP32 in *ocp-ko1* and TP309 were assessed by qRT-PCR (mean ± SD, n = 3). This experiment was repeated twice, ** P ≤ 0.01, *t* test. **(E)** Detection of the influence of OCP on the stability of OsRACK1A and OsSNAP32. EGFP-His served as the negative control. GST-OsRACK1A and GST-OsSNAP32 were detected with Anti-GST.

To clarify the co-expression of OCP and its interacting proteins, subcellular localization assays were carried out. The results indicated that OsRACK1A was located in the cytoplasm, agreeing with a previous study (Zhang et al., 2018), whereas the OsSNAP32 was located in the cell membrane as well as the cytoplasm (**Figure 5A**). Subcellular co-localization showed that both OCP and OsRACK1A or OsSNAP32 are expressed in the cytoplasm (**Figure 5B**), which makes their interaction possible. Then the CDS of *OsSNAP32* was joined to pGBKT7 (BD-OsSNAP32) and co-transformed into yeast cells with AD-OsRACK1A *via* the Y2H system. These results indicated a non-interaction between OsRACK1A and OsSNAP32. Yet OCP interacted separately with both OsRACK1A and OsSNAP32, raising the question, could they form a complex? To answer this, yeast three-hybrid (Y3H) assays were conducted. *OsSNAP32* and *OCP* were respectively inserted into the multiple cloning site (MCS) 1 (pBridge-OsSNAP32) and MCS2 (pBridge-OCP) of the Y3H vector pBridge, and also simultaneously (pBridge-OsSNAP32-OCP). We transformed the combinations with AD-OsRACK1A to yeast cells, which did not grow well on the SD/-Ade-His-Leu-Met-Trp medium. The results indicated that OsRACK1A and OsSNAP32 could not interact with each other when OCP acted as a bridge in the Y3H system (**Figure 5C**). Analyzing the transcript levels by qRT-PCR, we found that *OsSNAP32* mRNA accumulated to a significantly higher level (~10-fold) in *ocp-ko1* than in TP309, whereas the changed transcript levels of *OsRACK1A* was not significant (**Figure 5D**). We then examined the effect of OCP on the stability of OsRACK1A and OsSNAP32, finding it similar between the control and corresponding treatment, suggesting that OCP did not affect the stability of OsRACK1A and OsSNAP32 (**Figure 5E**). These results collectively show that OCP cannot form a complex with OsRACK1A and OsSNAP32, nor does it affect their stability, but it can suppress the expression of *OsSNAP32*.

### Analysis of differentially expressed genes related to the immune response between *ocp-ko1* and TP309

**Figure 6:**
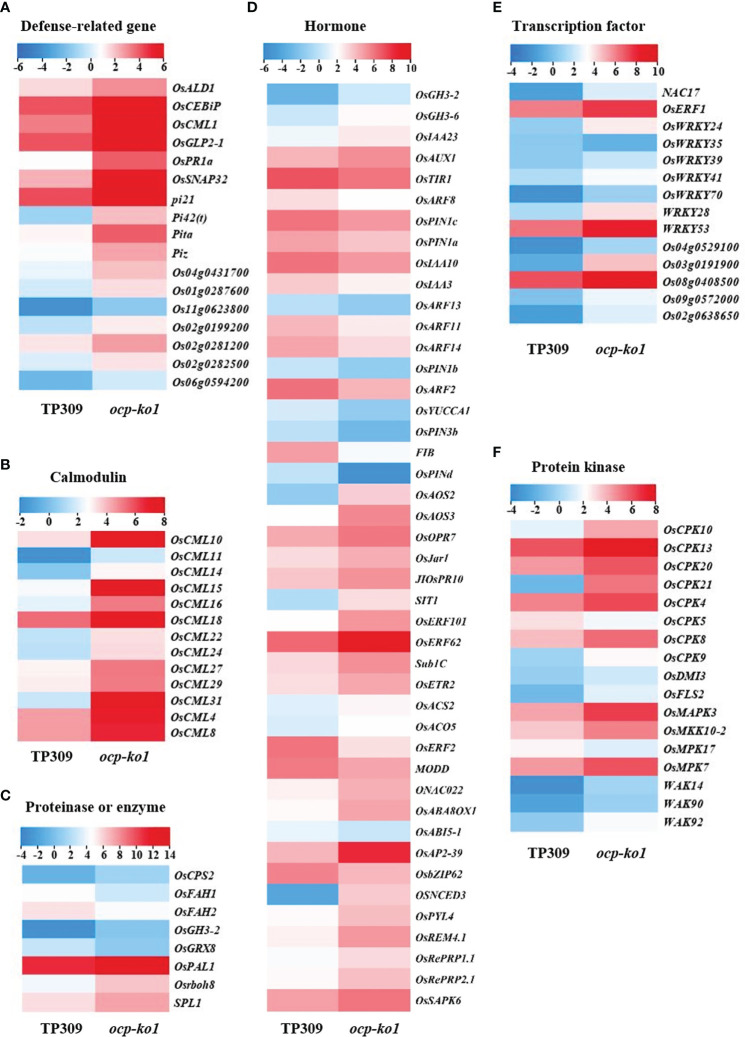
Detailed analysis of differentially expressed genes (DEGs) related to disease resistance in rice between *ocp-ko1* and TP309. **(A–F)**, heat map of DEGs. **(A)** Defense-related gene. **(B)** Calmodulin. **(C)** Proteinase or enzyme. **(D)** Hormone. **(E)** Transcription factor. **(F)** Protein kinase. The color legend insets indicate the log_2_ (FPKM) value.

To confirm *OCP*’s participation in the immune response of rice, we conducted RNA sequencing assays. The sequencing and mapping data are summarized in **Supplementary Table 4**. A total of 6956 DEGs were identified in the transcriptional profiles (having a fold-change ≥ 2 and p-adjusted value < 0.05), of which 4169 DEGs were up-regulated and 2787 DEGs were down-regulated in *ocp-ko1* vis-à-vis TP309 (**Supplementary Figure 7**). KEGG enrichment analysis revealed that the up-regulated DEGs were chiefly involved in phenylpropanoid biosynthesis, plant–pathogen interaction, flavonoid biosynthesis, MAPK signaling pathway-plant, and so on (**Supplementary Figure 8**). The down-regulated DEGs were enriched in terms of ribosome, DNA replication, plant hormone biosynthesis, signal transduction, and so on (**Supplementary Figure 9**). We found 71 DEGs responsive to plant-pathogen interaction and 47 DEGs functioning in the MAPK signaling pathway (**Supplementary Figure 8**), which are related to plant tolerance of biotic stress (Xiong and Yang, 2003; Chang et al., 2022). We divided these DEGs into several categories, namely those related to disease, transcription factor, calmodulin, proteinase, hormone, and protein kinase (**Figures 6A–F**).

Phytohormones play pivotal roles in plant defense responses. In this study, we identified some genes related to jasmonic acid (JA), ethylene (ET), auxin, and abscisic acid (ABA) (**Figure 6D**). *OsAOS2* (*allene oxide synthase 2*), *OsAOS3* (*allene oxide synthase 3*), *OsOPR7* (*OPDA reductase 7*), and *JIOsPR10* (*jasmonate inducible pathogenesis-related class 10*), all of which figure prominently in the biosynthesis of JA, were up-regulated dramatically, which could be viewed as resistance response to pathogens (Wang et al., 2021). *SIT1* (*salt intolerance 1*), *OsERF101* (*ethylene response factor 101*), *OsERF62* (*ethylene response factor 62*), *Sub1C* (*submergence1C*), *OsETR2* (*ethylene response 2*), *OsACS2* (*ACC synthase 2*), and *OsACO5* (*ACC oxidase 5*), which participate in ethylene (ET) biosynthesis and response (Helliwell et al., 2016), were increased significantly at the transcript level. Auxin response factors (*OsARF2*, *auxin response factor 2*; *OsARF11 auxin response factor 11*; etc.) and auxin efflux transporters (*OsPIN1a*, *pin-formed1a*; *OsPIN1d*, *pin-formed1d*; etc.) were strongly down-regulated; in stark contrast, the auxin influx carrier *OsAUX1* (*auxin transporter 1*) and IAA synthetase gene *OsGH3-2* (*gretchen hagen 3-2*) were both up-regulated. Inducing the expression of *OsGH3-2* is known lower the auxin content, leading to an auxin deletion phenotype and enhanced resistance to rice blast (Fu et al., 2011). Concerning ABA (Spence et al., 2015), some genes related to it were up-regulated; for instance, *ONAC022* (*NAC domain-containing protein 022*), *OsABA8OX1* (*ABA 8’-hydroxylase 1*), *OsAP2-39* (*APETALA-2-like transcription factor*), *OsNCED3* (*9-Cis-epoxycarotenoid dioxygenase 3*), and *OsREM4.1* (*remorin group 4 member 1*) were found up-regulated, while others were down-regulated, namely *MODD* (*mediator of OsbZIP46 deactivation and degradation*), *OsbZIP62* (*bZIP transcription factor 62*), and *OsABI5-1* (*abscisic acid insensitive 5*). Some genes can respond to more than one hormone at once; for example, *OsERF2* (*ethylene responsive factor 2*) (Xiao et al., 2016), an ethylene response factor, was evidently required for the control of the ET- and ABA-responses with its transcripts declining in *ocp-ko1*. The transcript levels of *pi21*, *Pi42(t)* (*Magnaporthe grisea resistance-42(t)*), *Pita* and *Piz* (*Magnaporthe grisea resistance-z*), the executive genes of rice blast resistance, were substantially increased (**Figure 6A**). Most transcription factors (*NAC17*, *NAC domain-containing protein 17*; *OsWRKY53*, *WRKY gene 53*; etc.), proteinases (*OsPAL1*; *SPL1*, *sphingosine-1-phosphate lyase 1*; etc.), and protein kinases (*OsFLS2*, *flagellin sensitive 2*; *OsMAPK3*, *mitogen-activated protein kinase*; etc.) were up-regulated in *ocp-ko1*, when compared with TP309 (**Figures 6C, E, F**). Intriguingly, the transcript levels of many calmodulin genes (*OsCML4*, *calmodulin-like 4*; *OsCML8*; *OsCML10*; etc.) and calcium-dependent protein kinase genes (*OsCPK4*, *calcium-dependent protein kinase 4*; *OsCPK5*; *OsCPK8*; etc.) were drastically increased (**Figures 6B, F**). These results suggested that *OCP* could regulate blast resistance by influencing the expression of defense-related genes.

**Figure 7:**
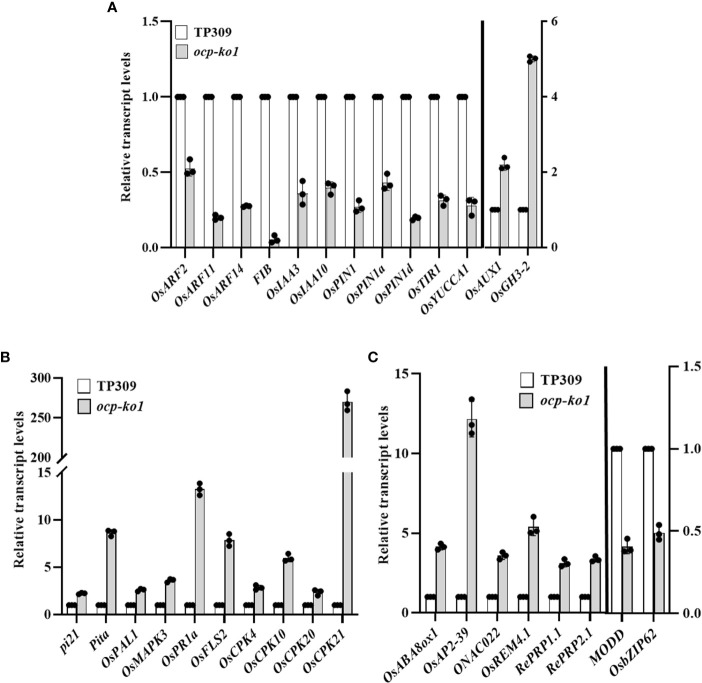
Relative transcript levels of (DEGs) related to disease resistance in rice between *ocp-ko1* and TP309 validated by qRT-PCR. **(A)** Relative transcript levels of auxin-related genes. **(B)** Relative transcript levels of blast-related genes. **(C)** Relative transcript levels of ABA-related genes (mean ± SD, n = 3). This experiment was repeated twice.

To verify the RNA sequencing results of *ocp-ko1* and TP309, we designed primers for qRT-PCR to compare the turnover rate of genes’ mRNA related to blast (**Figures 7A–C**). All tested genes presented a difference and reached a significant level. The response of many genes response to auxin changed significantly (**Figure 7A**). The blast resistance genes *pi21* and *Pita* were up-regulated more than twice and eight-fold, respectively. The mRNA of *OsPAL1* and *OsMAPK3*, which enhance resistance to blast when overexpressed, accumulated in *ocp-ko1*. Both *OsPR1a* (*pathogenesis-related 1a*) and *OsFLS2*, which trigger an immune response in response to pathogen inoculation, were up-regulated about 13 and 8 times, respectively. Moreover, *OsCPK4*, *OsCPK10*, *OsCPK20*, and *OsCPK21*, all of which encode calcium-dependent protein kinases, were expressed more in *ocp-ko1*, and these genes’ up-regulation conferred enhanced immunity upon pathogen infection (**Figure 7B**). Finally, *OsABA8ox1*, *OsAP2-39*, and *OsREM4.1* were up-regulated while both *MODD* and *OsbZIP62* were down-regulated (**Figure 7C**). The above qRT-PCR results were consistent with those of the transcriptome analysis, which suggested OCP may regulate the blast resistance by affecting various pathways in rice.

## Discussion

Our study focused on the PLCP gene *OCP*, which was involved in regulating the rice response to the blast-causing fungus *M. oryzae*. We selected three loci distributed in different domains of *OCP* for editing, and the pathogen inoculation results showed that the *OCP* knockout lines presented resistance to blast isolates 97-27-2, JL021605, and ZB13, whereas the *OCP* overexpression lines and TP309 did not and responded similarly (**Figures 2C-F**). OCP possessed cysteine protease activity, and it interacted with OsRACK1A and OsSNAP32 physically *in vitro* and *in vivo*. Accordingly, it is worthwhile to study the genetic relationship between OCP and OsRACK1A or OsSNAP32. Further, *OCP* influenced the expression of some genes related to blast resistance.

### *OCP* has pleiotropic effects on rice development and resistance

In the study, *ocp-ko* lines showed increased resistance to *M. oryzae*, but not to *Xoo*, whereas *OCP-OE* was susceptible to both phytopathogens. Therefore, OCP negatively influences plant defense against fungal pathogens. In Arabidopsis, the *rd21* null mutants were more susceptible to the necrotrophic fungal pathogen (Shindo et al., 2012); conversely, null *XCP1* or *XCP2* mutants displayed enhanced resistance (Zhang et al., 2014; Perez-Lopez et al., 2021). Therefore, genes harboring the same functional domains do not necessarily function in the same way.

The homologous gene *RD21* of *OCP* in *A. thaliana* responds to biotic and abiotic stressors (Kikuchi et al., 2008; Rustgi et al., 2017), and *OsCP1* in rice is known to affect pollen development (Lee et al., 2004). Here, we found that knocking out *OCP* led to shorter plant height and lower fertility than TP309. The plant height of *ocp-ko1* was significantly reduced to 103.9 ± 0.89 cm, while TP309 was taller, at 119.5 ± 0.79 cm (**Supplementary Figure 10**). Compared with TP309, the panicle length of *ocp-ko1* was shorter and it had more empty grains, mainly due to the abnormal pollen development of *ocp-ko1* that resulted in its significantly decreased seed setting rate (**Supplementary Figure 11**). Therefore, OCP is a pleiotropic gene, which modulated blast resistance yet also influenced plant height and pollen development. In further research, we will aim to identify the mechanism by which OCP regulates plant height and fertility.

### *OCP* negatively regulates blast resistance *via* multiple pathways

PLCPs play key roles in the growth and development of plants, as well as the immune responses to pathogens (Avci et al., 2008; Liu et al., 2018). Yet, further investigation is required to uncover the protease substrates and functional pathways (Demir et al., 2018). We found OCP located in the cytoplasm interspersed with OsRACK1A and OsSNAP32 (**Figure 5A**); not surprisingly, perhaps, these two proteins physically interacted with OCP (**Figure 4**). Nevertheless, the yeast hybrid results showed that OsRACK1A and OsSNAP32 did not interact with each other, and they could not form a ternary complex with OCP (**Figure 5C**). *In vitro*, we proved that OCP possessed cysteine protease activity (**Figure 3D**). Expression analysis found that many genes related to disease resistance, such as *pi21*, *Pi42(t)*, *OsSNAP32*, and *OsMAPK3* (among others), were up-regulated in *ocp-ko1*. Therefore, it is quite plausible that *OCP* negatively regulates blast resistance by influencing the expression of *OsSNAP32* and other disease-resistance genes.

During immune responses, phytohormones act as signals to trigger and mediate defense responses in plants against enemies (Yang et al., 2013). JA functions critically in the basal defense of rice, especially against necrotrophic pathogens (Browse, 2009). ET regulates disease resistance positively or negatively depending on the different pathogens and local environmental conditions (Broekaert et al., 2006; van Loon et al., 2006), and exogenous application of an ET generator could increase rice blast disease resistance (Singh et al., 2004). Auxin, being a widespread important hormone in plants, is involved in almost all developmental processes. The accumulation of auxin content in the model plants Arabidopsis and rice leads to their increased susceptibility to disease (Yang et al., 2013). Regarding ABA, its application to rice suppresses resistance to blast (Koga et al., 2004; Yang et al., 2013). In our study, the expression of particular genes known to participate in hormone synthesis and metabolism was changed in *ocp-ko1* plants (**Figure 6B**, **Figures 7A, C**). Often, hormones interplay and engage in hormonal crosstalk to defend plants against pathogens (Kazan and Lyons, 2014). In addition to the above, many other DEGs, transcription factors, protein kinases, and so forth, were proven to respond to *M. oryzae*. Importantly, some calmodulin genes were up-regulated significantly; hence, it is possible that *OCP* suppresses blast resistance *via* multiple pathways, wherein calmodulin might play a crucial role. To better understand the molecular mechanisms of OCP-mediated blast resistance in rice, further investigations are needed to clarify the signaling pathways of *OCP* vis-à-vis other factors in the plant immune response.

## Conclusion

OCP negatively regulates blast resistance in rice, because all *ocp-ko1*, *ocp-ko2*, and *ocp-ko3* lines have enhanced resistance to *M. oryzae*. OCP is expressed in all rice tissues and located mainly in the cytoplasm, interacting with OsRACK1A and OsSNAP32 *in vivo* and *in vitro*, but they could not form a complex. The transcriptome analysis shows that the expression of many factors responsive to *M. oryzae* are changed in *ocp-ko1* significantly, including the phytohormones JA, ET, auxin, and ABA, which suggests that OCP could affect host resistance to rice blast in multiple ways and plays a fundamental role. Therefore, this study’s findings provide the basis for exploring the molecular mechanism of cysteine protease in the disease resistance of rice. Further, in screening many potential interaction proteins of OCP, this work can assist in comprehensively investigating the effects of *OCP* on rice growth and development.

## Data availability statement

The original contributions presented in the study are publicly available. This data can be found here: NCBI, PRJNA855166. Sequence data from this article can be found in the GenBank database under the following accession numbers: OCP, LOC_Os04g55650; OsRACK1A, Os 0 1 g 0 6 8 6 8 0 0 ; OsSNAP32, Os02g0437200.

## Author contributions

YL, WZ, and CL conceived the study and designed the experiments. YL, PL, LM, GJ, and QL performed the experiments. YL and CL wrote the manuscript. All authors contributed to the article and approved the submitted version.

## Funding

This work was supported by the National Natural Science Foundation of China (grant nos. 31900383, 31971911), and China Postdoctoral Science Foundation (grant no. 2019M660853).

## Acknowledgments

We thank Dr. Zhuangzhi Zhou and Dr. Minxiang Yu for supplying the *M. oryzae* isolates 97-27-2, JL021605 and ZB13.

## Conflict of interest

The authors declare that the research was conducted in the absence of any commercial or financial relationships that could be construed as a potential conflict of interest.

## Publisher’s note

All claims expressed in this article are solely those of the authors and do not necessarily represent those of their affiliated organizations, or those of the publisher, the editors and the reviewers. Any product that may be evaluated in this article, or claim that may be made by its manufacturer, is not guaranteed or endorsed by the publisher.
