# Supplementary material for: Knockout of a papain-like cysteine protease gene OCP enhances blast resistance in rice
Source: Front Plant Sci. 2022 Nov 30;13:1065253. doi: 10.3389/fpls.2022.1065253 (PMC9749133; doi:10.3389/fpls.2022.1065253)
Supplement: Supplementary file 2 [file DataSheet_2.docx]

Supplementary Data


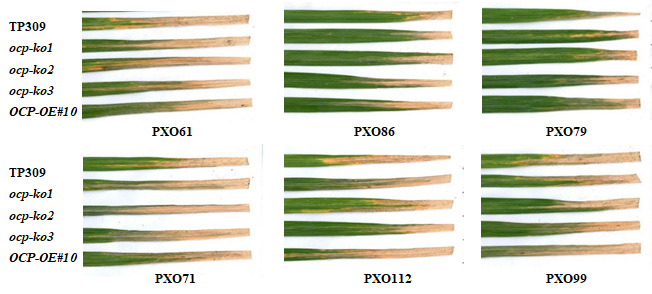


Supplementary Figure 1 Leaf phenotypes of TP309 and *OCP* mutant lines inoculated with *Xoo* isolates

TP309: wild type. *ocp-ko1*, *ocp-ko2*, *ocp-ko3*: *OCP* knockout lines. *OCP-OE#10*: *OCP* overexpression line. PXO61, PXO86, PXO79, PXO71, PXO112, PXO99: *Xanthomonas oryzae* pv*. oryzae* (*Xoo*) isolates.


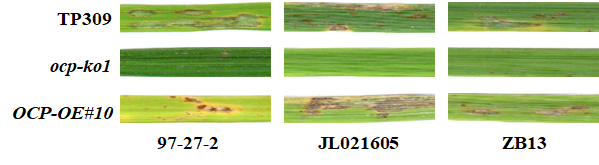


Supplementary Figure 2. Spray inoculation of TP309 and *OCP* mutant lines with the blast isolate 97-27-2, JL021605 and ZB13


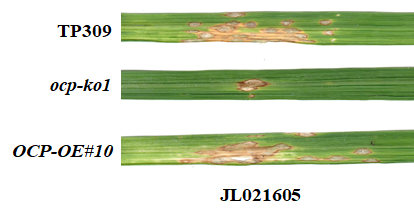


Supplementary Figure 3. Injection inoculation of TP309 and *OCP* mutant lines with the blast isolate JL021605 in the field


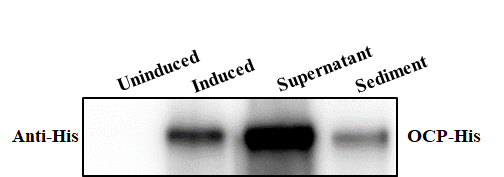


Supplementary Figure 4. Expression of OCP in *E.coli*.


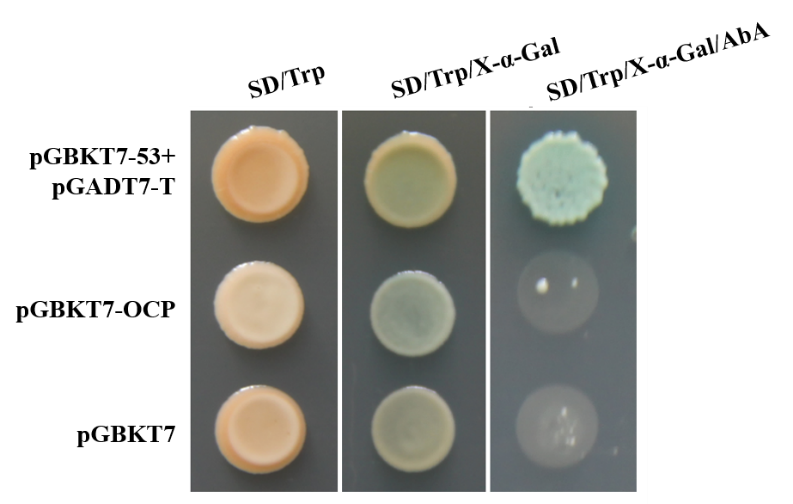


Supplementary Figure 5. Autoactivation and toxicity detection of OCP


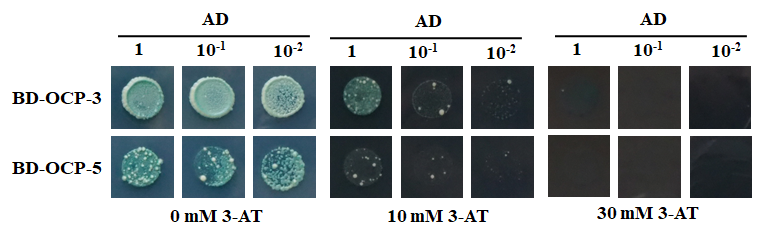


Supplementary Figure 6. 3-AT inhibited the interaction of BD-OCP-3 and BD-OCP-5 with the empty vector AD


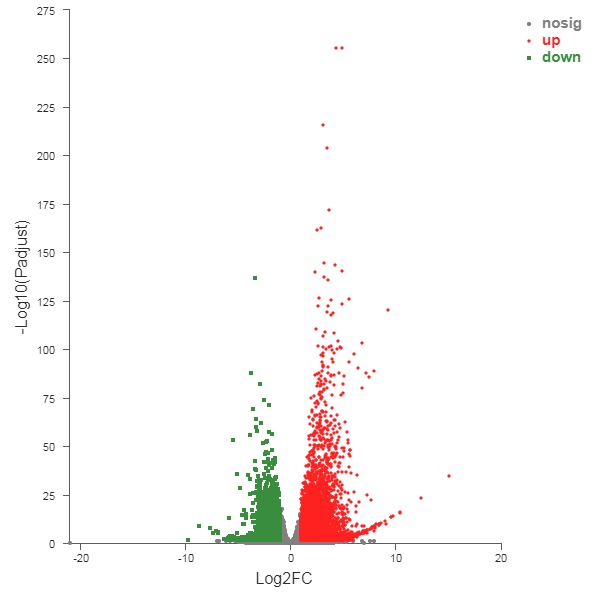


Supplementary Figure 7. Volcano plot of DEGs between *ocp-ko1* and TP309


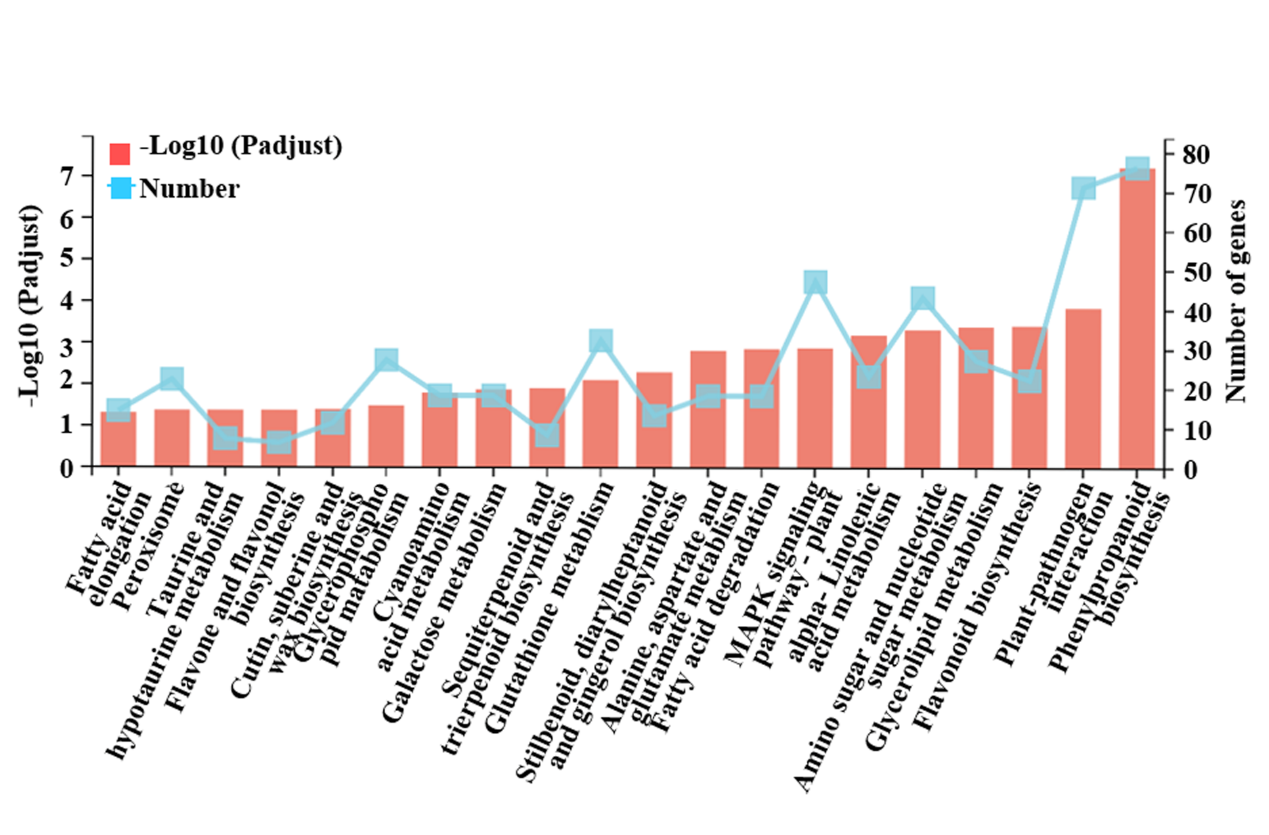


Supplementary Figure 8. Terms clustering analysis of up-regulated DEGs


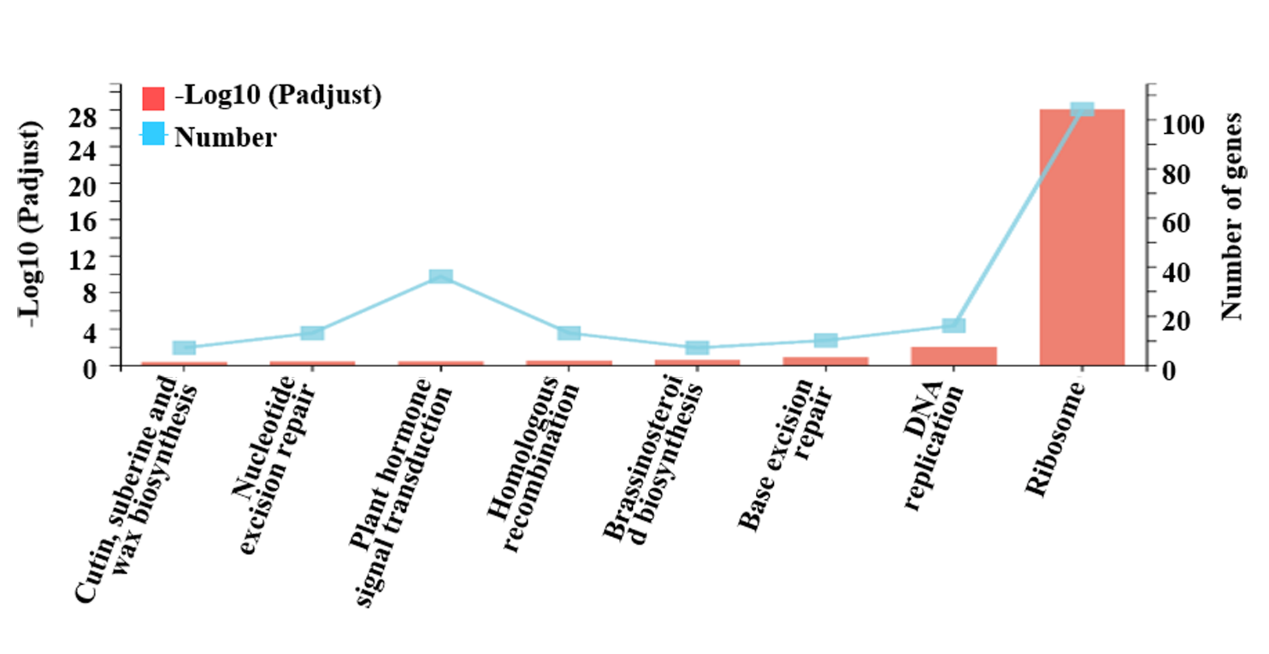


Supplementary Figure 9. Terms clustering analysis of down-regulated DEGs


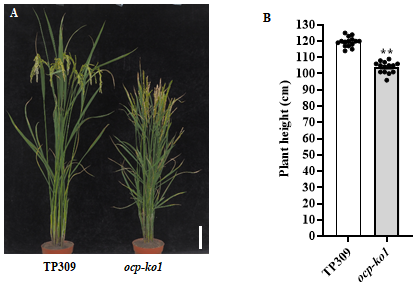


Supplementary Figure 10. Comparison of plant height between TP309 and *ocp-ko1*

(A) Comparison of plant morphology between TP309 and *ocp-ko1*. Bar = 10 cm. (B) the plant height of TP309 and *ocp-ko1*. Dots represent raw data, mean ± SEM, n = 15.


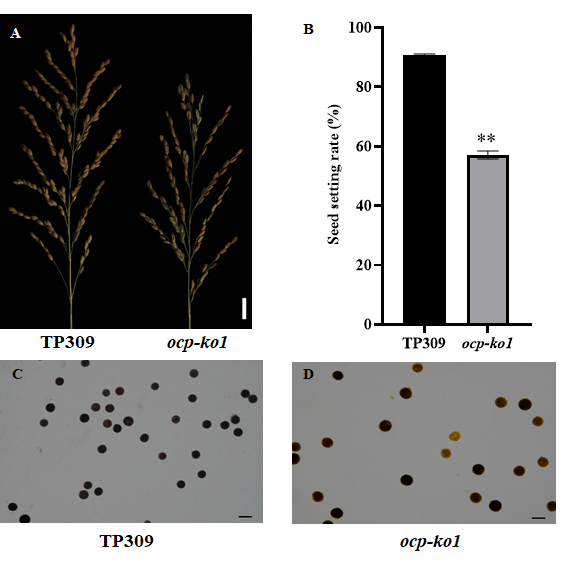


Supplementary Figure 11. Comparison of fertility between TP309 and *ocp-ko1*

(A) The panicle morphology of TP309 and *ocp-ko1*. Bar = 2 cm. (B) Seed setting rate of *ocp-ko1*. n =15. (C) and (D) Pollen staining results of TP309 and *ocp-ko1*, respectively. Bar = 50 μm.

Supplementary Table 1. Primers used in this study for vector constructions

| **Primer name** | **Sequence (5'-3')** | | | |  |
| --- | --- | --- | --- | --- | --- |
| AD-OCPF | GGAGGCCAGTGAATTCATGAGGATTTCCATGGCTCT | | | |  |
| AD-OCPR | TCTGCAGCTCGAGCTCTCAAGCGCTGCTCTTCTTGC | | | |  |
| AD-OsRACK1AF | GGAGGCCAGTGAATTCATGGCCGGCGCGCAGGAGTC | | | |  |
| AD-OsRACK1A R | TCTGCAGCTCGAGCTCCTAGCCGGCGTAGCTGAAACCT | | | |  |
| AD-OsSNAP32F | GGAGGCCAGTGAATTCATGTGCTGCTCTTCCTTGTTC | | | |  |
| AD-OsSNAP32R | TCTGCAGCTCGAGCTCTTATTTTCCAAGCAGACGGCG | | | |  |
| BD-OCPF | GCCATGGAGGCCGAATTCATGAGGATTTCCATGGCT | | | |  |
| BD-OCPR | GTTATGCGGCCGCTGCAGTCAAGCGCTGCTCTTCTT | | | |  |
| BD-OCP-1R | GTTATGCGGCCGCTGCAGCTTGTTCCTGAGGCCGAG | | | |  |
| BD-OCP-2F | GCCATGGAGGCCGAATTCCCGCGGAGGGAGAGGAAG | | | |  |
| BD-OCP-2R | GTTATGCGGCCGCTGCAGCTCGCCCTTCTTCAGAGG | | | |  |
| BD-OCP-3F | GCCATGGAGGCCGAATTCAACCCCCCGAACCCCGGC | | | |  |
| BD-OCP-3R | TCTGCAGCTCGAGCTCCTTGTTCCTGAGGCCGAG | | | |  |
| OCP-MCS2F | AGAAAGGTGGCGGCCGCAATGAGGATTTCCATGGCTCT | | | |  |
| OCP-MCS2R | AGATCAGCCCGAAGATCTTCAAGCGCTGCTCTTCTTGCC | | | |  |
| OsRACK1A-MCS1F | ACTGTATCGCCGGAATTCATGGCCGGCGCGCAGGAGTC | | | |  |
| OsRACK1AMCS1R | TGCAGGTCGACGGATCCCCTAGCCGGCGTAGCTGAAAC | | | |  |
| OsSNAP32-MCS1F | ACTGTATCGCCGGAATTCATGTGCTGCTCTTCCTTGTTC | | | |  |
| OsSNAP32-MCS1R | TGCAGGTCGACGGATCCCTTATTTTCCAAGCAGACGGC | | | |  |
| cYFP-OCPF | | | CCCAGGCCTACTAGTGGAATGAGGATTTCCATGGCTCT | | |
| cYFP-OCPR | | | CTCCTACCCGGGAGCGGTTCAAGCGCTGCTCTTCTTGCC | | |
| nYFP–OsRACK1AF | | | CCCAGGCCTACTAGTGGAATGGCCGGCGCGCAGGAGTC | | |
| nYFP-OsRACK1AR | | | CTCCTACCCGGGAGCGGTCTAGCCGGCGTAGCTGAAACCT | | |
| nYFP-OsSNAP32F | | | CCCAGGCCTACTAGTGGAATGTGCTGCTCTTCCTTGTTC | | |
| nYFP-OsSNAP32R | | | CTCCTACCCGGGAGCGGTTTATTTTCCAAGCAGACGGCG | | |
| OCP-MycF | | | GGTCGACTCTAGAGGATCCGAATTCGGCATGAGGATTTCCATGGCTCTC | | |
| OCP-MycR | | | ACCGGTTCCACCTCCGGAAGCGCTGCTCTTCTTGCC | | |
| OCP-HisF | | | AGCAAATGGGTCGCGGATCCATGAGGATTTCCATGGCTCTC | | |
| OCP-HisR | | | CGAGTGCGGCCGCAAGCTTAGCGCTGCTCTTCTTGCC | | |
| GST-OsRACK1AF | | | TGGTGGTGGTGGAATTCTAATGGCCGGCGCGCAGGAGTC | | |
| GST-OsRACK1AR | | | GTCACGATGAATTAAGCTTCTAGCCGGCGTAGCTGAAAC | | |
| GST- OsSNAP32F | | | TGGTGGTGGTGGAATTCTAATGTGCTGCTCTTCCTTGTTC | | |
| GST- OsSNAP32R | | | GTCACGATGAATTAAGCTTTTATTTTCCAAGCAGACGGC | | |
| OCP-eYFPF | | ATCTCGAGCTCAAGCTTCATGAGGATTTCCATGGCTCTC | |  |  |
| OCP-eYFPR | | CCGCGGTACCGTCGACTAGCGCTGCTCTTCTTGCC | |  |  |
| OCP-GFPF | | CACGGGGGACTCTAGATGAGGATTTCCATGGCTCTC | |  |  |
| **Primer name** | | **Sequence (5'-3')** | |  |  |
| OCP-GFPR | | CCTTACCCATGGTACCAGCGCTGCTCTTCTTGCC | |  |  |
| OsRACK1A-mCherryF | | ATCTCGAGCTCAAGCTTATGGCCGGCGCGCAGGAGTC | |  |  |
| OsRACK1A-mCherryR | | GCTCACCATCAGGATCCCGCCGGCGTAGCTGAAACCT | |  |  |
| OsSNAP32-mCherryF | | ATCTCGAGCTCAAGCTTATGTGCTGCTCTTCCTTGTTC | |  |  |
| OsSNAP32-mCherryR | | GCTCACCATCAGGATCCCTTTTCCAAGCAGACGGCG | |  |  |

Supplementary Table 2. The qRT-PCR primers used in this study

| **Primer name** | **Sequence (5'-3')** |
| --- | --- |
| RT-OCPF | AACCTGCCTCATGGCTAAGG |
| RT-OCPR | CTTCTTGCCGTTGCCAAACA |
| RT-OsYUCCA1F | GAAGGTGTTGGTCGTGGGAT |
| RT-OsYUCCA1R | GGTAGGACATGCACCGTGTT |
| OsActinF | CCTGACGGAGCGTGGTTAC |
| OsActinR | CCAGGGCGATGTAGGAAAGC |
| RT-FIBF | CTTCTGCAACTTCACCAAGGAG |
| RT-FIBR | AATCCTCCACATCCTCCCTATC |
| RT-OsGH3-2F | TGATCACTCACTACACTACACG |
| RT-OsGH3-2R | ACACTGACACCGACTGTATAAG |
| RT-OsTIR1F | GAGATCATGAACGATCCTGGAA |
| RT-OsTIR1R | CGGTAGACGTACAGTTTCTCAA |
| RT-OsPIN1dF | TGATCAGGAACCCAAACACTTA |
| RT-OsPIN1dR | GCATCTGAAAGGATCGAAATCG |
| RT-OsMAPK3F | TTACCTAGAGAGATTGCACGAC |
| RT-OsMAPK3R | TGGTGATTCAATCTAGTACCGG |
| RT-OsREM4.1F | TGTGGAACTCTCGTTTTAGTGT |
| RT-OsREM4.1R | TTACTTGTTCTGTCTGTCCCTC |
| RT-OsABA8OX1F | CGGTATGGGTTTTCCTGACC |
| RT-OsABA8OX1R | CTCCCTGTTCGGTTTTCACTC |
| RT-ONAC022F | AAGAAGGAAGAAGCAGACGTC |
| RT-ONAC022R | CTTAGTAGTAGAGCAGATGGGC |
| RT-REPRP1.1F | AAGCTCACAGTTCAGTTACGTA |
| RT-REPRP1.1R | TTCATGCTTAGGATGAGGCTTT |
| RT-REPRP2.1F | GTTGTTTGCTACGTATGATCCC |
| RT-REPRP2.1R | GAACAACACACGAAAAACACAC |
| RT-MODDF | GGAAATGTGAACCATAGCAGTG |
| RT-MODDR | TATGCTTTGTTGGTATTCCGGA |
| RT-OsBZIP62F | GATTCTCTTCTCATCTGGTCCC |
| RT-OsBZIP62R | CACACACACTCACCCTACC |
| RT-OsRACK1AF | TGTGGAACCTCACGAACTG |
| **Primer name** | **Sequence (5'-3')** |
| RT-OsRACK1AR | AAGCGAGTACAGCCTCTTG |
| RT-OsSNAP32F | CCAATTCAGTTTCATTTGTGCG |
| RT-OsSNAP32R | TTCTTGGATGCGAAGAACGATC |
| RT-OsFLS2F | TGTACCTCCACTCCGGGTA |
| RT-OsFLS2R | GAACTCTGGAGCCATGTAACC |
| RT-OsCPK4F | TTGCTGTGGAGGATGTAAAGAG |
| RT-OsCPK4R | CAAACGCATTGTAGAAGTGAAC |
| RT-OsCPK10F | CAGAGAACGAGCAGGGTATATT |
| RT-OsCPK10R | AACAAGCATTCTCCTTACGAGA |
| RT-OsCPK20F | GTGAATTCCCCAGATGAAAACC |
| RT-OsCPK20R | ATAGAGCAGTGGAAATCTCGAG |
| RT-OsCPK21F | GAGATCAGGATGCTACTCGAAG |
| RT-OsCPK21R | GTCCTTGTCGAAGTAGTTGAAC |
| RT-PitaF | ATCGACTCCTTGAAACTATCC |
| RT-PitaR | AGTATTCTACTGCAACTATTATTATC |
| RT-pi21F | CGGCAAATTTGACAGATGGGTAT |
| RT-pi21R | CTTCTCCGGGTCGAACTTC |
| RT-OsPR1aF | GGCCAATCTCCCTACTGATTAA |
| RT-OsPR1aR | GCATAAACACGTAGCATAGCAT |
| RT-OsPAL1F | GACCCTGTATTTTCTTCGTTCG |
| RT-OsPAL1F | AGTAGCAATACTTTCACCCCAA |
| RT-OsIAA3F | GGCCATGTTCCTCTGCTTCT |
| RT-OsIAA3R | GCACGTACTAATGAACATCTCGAA |
| RT-OsIAA10F | GATGGTGACTGGATGCTCGT |
| RT-OsIAA10R | ATCGCTGACCAAGACCATTCG |
| RT-OsAUX1F | CATCACCCACACCGGTCCAA |
| RT-OsAUX1R | CGTGCATGATCTCCACTGTGAC |
| RT-OsAP2-39F | AGCGACGTGTAATAGTAAGAGG |
| RT-OsAP2-39R | AAGGACACGATCTATGTACACC |
| RT-OsARF14F | CAAGGAGTGGTGCGTCAGAA |
| RT-OsARF14R | CCAGCGGACCTCTCATTAGC |
| RT-OsARF2F | TCGAGAGACCGACCACAGAAT |
| RT-OsARF2R | GCATGCGGTCTAATCACTCTTG |
| RT-OsARF11F | CAGCCTGTCATTGATTCGATTT |
| RT-OsARF11R | TACTGTTTTGCTCCGAAGTACT |
| RT-OsPIN1aF | TGCACCCCGACATTCTCA |
| RT-OsPIN1aR | GGATGTAGTACACCAGCGTGATG |
| RT-OsPIN1F | CTGAGCTGAGCTGTGAAATAGT |
| RT-OsPIN1R | GTCATCACGTGGTAGAAGTCC |

Supplementary Table 3. Screening of proteins interacting with OCP via the yeast two-hybrid system

| **Order** | **Gene locus** | **Gene symbol** | **Information** |
| --- | --- | --- | --- |
| 1 | *Os11g0523800* | *ARF1;OsARF23* | Auxin response factor |
| 2 | *Os06g0622500* | *CDC27* | Cell division cycle protein 27 homolog B |
| 3 | *Os01g0940000* | *CKX4 ; REN1* | Cytokinin oxidase/dehydrogenase family gene |
| 4 | *Os04g0563700* | *CYCB2;1* | Cyclin; cell cycle-related gene |
| 5 | *Os07g0129700* | *OSH15;Oskn3* | KNOX family class 1 homeobox gene of rice |
| 6 | *Os01g0667400* | *DWT1; OsWOX9a* | DWARF TILLER1 |
| 7 | *Os01g0741900* | *IAA6* | Auxin-responsive Aux/IAA gene |
| 8 | *Os01g0831000* | *LAX1 ; bhlh123* | Lax panicle; HLH transcription factor |
| 9 | *Os04g0396500* | *LAX2 ; Gnp4* | Lax panicle2;Grain Number Per-Panicle Gene |
| 10 | *Os04g0663600* | *MOC3; TAB1* | Monoculm 3; Ortholog of WUSCHEL |
| 11 | *Os03g0727000* | *OSH1; Oskn1* | KNOX family class 1 homeobox gene of rice |
| 12 | *Os03g0727200* | *OSH3* | KNOX family class 1 homeobox gene of rice |
| 13 | *Os03g0771500* | *OSH43* | KNOX family class 1 homeobox gene of rice |
| 14 | *Os12g0274700* | *OsRBCS2* | Small subunit of Rubisco |
| 15 | *Os02g0437200* | *OsSNAP32* | Soluble N-ethylmaleimide-sensitive factor attachment protein receptor |
| 16 | *Os08g0509600* | *OsSPL14; IPA1* | Squamosa promoter binding protein-like 14 |
| 17 | *Os03g0706500* | *OsTB1; FC1* | TCP family transcription factor |
| 18 | *Os03g0599800* | *OsUAM1* | UDP-arabinopyranose mutase 1 |
| 19 | *Os01g0686800* | *RWD; OsRACK1A* | Receptor for activated C kinase 1 |
| 20 | *Os03g0123300* | *TAD1; TE* | Tillering and Dwarf 1; Tiller Enhancer |
| 21 | *Os05g0150500* | *TIR1* | Auxin receptor |
| 22 | *Os03g0182600* |  | Ribosomal protein S2, putative, expressed |
| 23 | *Os06g0686400* |  | LTPL85-Protease inhibitor/seed storage/ LTP family protein precursor, expressed |
| 24 | *Os07g0556200* |  | Cytochrome b6-f complex iron-sulfur subunit, chloroplast precursor, putative, expressed |
| 25 | *Os03g0756400* |  | Expressed protein |
| 26 | *Os02g0103700* |  | Ribosomal protein L6, putative, expressed |
| 27 | *Os12g0180400* |  | RNA polymerases N 8 kda subunit, putative, expressed |
| 28 | *Os01g0550300* |  | N-rich protein, putative, expressed |
| 29 | *Os01g0659200* |  | Vacuolar ATP synthase subunit E, putative, expressed |
| 30 | *Os04g0105200* |  | Expressed protein |
| 31 | *Os05g0110600* |  | Rab5-interacting protein like, putative, expressed |
| 32 | *Os08g0562800* |  | MATE efflux family protein, putative, expressed |
| 33 | *Os02g0175800* |  | Fiber protein Fb15, putative, expressed |
| 34 | *Os08g0560900* |  | Photosystem I reaction center subunit II, chloroplast precursor, putative, expressed |
| 35 | *Os06g0142300* |  | Early nodulin 93 ENOD93 protein, putative, expressed |
| 36 | *Os02g0179100* |  | HD domain containing protein 2, putative, expressed |
| 37 | *Os06g0601100* |  | Expressed protein |
| 38 | *Os02g0246300* |  | Prefoldin subunit, putative, expressed |
| 39 | *Os10g0363600* |  | Osfbk21-F-box domain and kelch repeat containing protein, expressed |
| 40 | *Os07g0640200* |  | FGGY family of carbohydrate kinases, putative, expressed |
| 41 | *Os04g0445200* |  | Enzyme of the cupin superfamily protein, putative, expressed |
| 42 | *Os07g0556200* |  | Cytochrome b6-f complex iron-sulfur subunit, chloroplast precursor, putative, expressed |
| 43 | *Os01g0501800* |  | Oxygen-evolving enhancer protein 1, chloroplast precursor, putative, expressed |
| 44 | *Os02g0131700* |  | Nuclear transport factor 2, putative, expressed |
